# Supplementary material for: Blood Pressure Patterns in Patients with Parkinson’s Disease: A Systematic Review
Source: J Pers Med. 2021 Feb 15;11(2):129. doi: 10.3390/jpm11020129 (PMC7918947; doi:10.3390/jpm11020129)
Supplement: Supplementary file 1 [file jpm-11-00129-s001.pdf]

Supplementary Materials:

Table S1. Blood pressure profiles.

No.	Study	HBP (no. of patients)	SH (no. of patients)	OH (no. of patients)	NH (no. of patients)	AntiHBP drugs (no. of patients)	Other VA Drugs (no. of patients)	LEDD (mg)
1	Arici and Helvaci (2020) [15]	-	-	-	-	-	-	954.2 ± 507.8
2	Chen et al. (2020) [16]	Excl.	35	26	63	Excl.	Excl.	277 ± 238.7
3	Di Stefano et al. (2020) [17]	Excl.	-	27	31	Excl.	Excl.	688.2
4	Oka et al. (2020) [18]	-	30	39	-	-	Excl.	Excl.
5	Yoo et al. (2020) [19]	43	-	33	45	-	Excl.	Excl.
6	Kotagal et al. (2019) [12]	-	-	-	-	-	-	835.98
7	Li et al. (2019) [20]	60	-	49	-	-	-	-
8	Vallelonga et al. (2019) [21]	32	-	53	49	33	30	693 ± 371
9	Vallelonga et al. (2019) [22]	15	41	61	-	-	-	712 ± 459
10	Milazzo et al. (2018) [23]	30	35	51	-	33	Excl.	698.7 ± 366.9
11	Tanaka et al. (2018) [24]	29	19	69	103	25	10	957.2 ± 360.1
12	Yamashiro et al. (2018) [25]	31	20	60	80	27	10	-
13	Franzen et al. (2017) [26]	16	-	10	14	7	-	839.08
14	Kanegusuku et al. (2017) [27]	Excl.	-	-	-	Excl.	Excl.	-
15	Kim et al. (2017) [28]	50	35	30	39	Excl.	Excl.	520.4 ± 264.2
16	Kim et al. (2017) [29]	42	39	32	31	-	-	-
17	Vetrano et al. (2017) [30]	115	-	-	-	-	-	8.1/kg
18	Vichayanrat et al. (2017) [31]	-	-	18	17	-	-	-
19	Kang et al. (2016) [32]	-	-	17	27	-	-	-
20	Kim et al. (2016) [33]	69	59	56	47	64	-	513.5 ± 302.4
21	Park et al. (2016) [34]	42	16	46	26	37	-	Excl.
22	Stuebner et al. (2015) [35]	Excl.	-	2	-	Excl.	Excl.	423 ± 431
23	Vetrano et al. (2015) [36]	76	-	-	-	-	-	8.3/kg
24	Fanciulli et al. (2014) [14]	-	3	4	1	4	0	-
25	Kim et al. (2014) [37]	16	15	30	8	-	-	Excl.
26	Oh et al. (2014) [38]	96	69	73	52	-	-	Excl.
27	Pilleri et al. (2014) [13]	-	-	-	15	21	2	969 ± 382.2
28	Berganzo et al. (2013) [39]	46	-	45	-	48	-	642.9 ± 349.9
29	Oh et al. (2013) [40]	36	-	-	27	-	-	Excl.
30	Oh et al. (2013) [41]	23	-	17	-	-	Excl.	Excl.
31	Kim et al. (2012) [42]	27	18	32	-	24	-	Excl.
32	Manabe et al. (2011) [43]	0	-	0	-	-	-	313.6
33	Sommer et al. (2011) [44]	21	11	21	25	21	-	-
34	Reimann et al. (2010) [45]	11	-	10	13	-	-	-
35	Schmidt et al. (2009) [46]	8	-	10	11	Excl.	Excl.	539 ± 211
36	Ejaz et al. (2006) [47]	4	-	-	13	3	-	-
37	Sigurdardóttir et al. (2001) [48]	-	-	-	-	-	3	1256
38	Plaschke et al. (1998) [49]	-	-	-	-	Excl.	Excl.	-
39	Senard et al. (1992) [50]	Excl.	-	19	-	Excl.	-	660
40	Micieli et al. (1989) [51]	0	-	1	-	0	0	0
	TOTAL	938 / 2460 (38.13%)	445 / 1603 (27.76%)	941 / 2433 (38.67%)	737 / 1894 (38.91%)	347 / 1169 (29.68%)	55 / 470 (11.7%)	

antiHBP = antihypertensive, Excl. = exclusion criterion, HBP = high blood pressure, LEDD = levodopa equivalent daily dose, OH = orthostatic hypotension, PD = Parkinson’s disease, SH = supine hypertension, VA = vasoactive.

Table S2. Dipping profiles.

No.	Study	No. of Patients	Reverse Dipping (< 0%)	Reduced Dipping (0-10%)	Normal Dipping (10-20%)	Extreme Dipping (> 20%)	Non-dipping (< 10%)	Dipping (> 10%)	Reduced Dipping & Dipping (> 0%)
1	Arici and Helvaci (2020) [15]	35	15	16	-	-	31	4	20
2	Chen et al. (2020) [16]	103	54	-	-	-	-	-	49
3	Di Stefano et al. (2020) [17]	52	26	14	9	3	40	12	26
4	Oka et al. (2020) [18]	75	-	-	-	-	38	37	-
5	Yoo et al. (2020) [19]	98	-	-	-	-	85	13	-
6	Kotagal et al. (2019) [12]	-	-	-	-	-	-	-	-
7	Li et al. (2019) [20]	-	-	-	-	-	-	-	-
8	Vallelonga et al. (2019) [21]	113	36	-	-	-	-	-	77
9	Vallelonga et al. (2019) [22]	72	54	-	-	-	-	-	18
10	Milazzo et al. (2018) [23]	114	37	37	33	7	74	40	77
11	Tanaka et al. (2018) [24]	137	52	51	27	7	103	34	85
12	Yamashiro et al. (2018) [25]	128	-	-	-	-	96	32	-
13	Franzen et al. (2017) [26]	22	18	-	-	-	-	-	4
14	Kanegusuku et al. (2017) [27]	-	-	-	-	-	-	-	-
15	Kim et al. (2017) [28]	125	-	-	-	-	99	26	-
16	Kim et al. (2017) [29]	99	-	-	-	-	81	18	-
17	Vetrano et al. (2017) [30]	-	-	-	-	-	-	-	-
18	Vichayanrat et al. (2017) [31]	51	15	15	-	-	30	21	36
19	Kang et al. (2016) [32]	46	13	20	-	-	33	13	33
20	Kim et al. (2016) [33]	188	-	-	-	-	153	35	-
21	Park et al. (2016) [34]	129	-	-	-	-	98	31	-
22	Stuebner et al. (2015) [35]	21	3	7	-	-	10	11	18
23	Vetrano et al. (2015) [36]	125	60	38	-	-	98	27	65
24	Fanciulli et al. (2014) [14]	16	1	4	-	-	5	11	15
25	Kim et al. (2014) [37]	65	-	-	-	-	52	13	-
26	Oh et al. (2014) [38]	225	-	-	-	-	180	45	-
27	Pilleri et al. (2014) [13]	61	34	16			50	11	27
28	Berganzo et al. (2013) [39]	111	26	53	26	6	79	32	85
29	Oh et al. (2013) [40]	129	-	-	-	-	102	27	-
30	Oh et al. (2013) [41]	69	-	-	-	-	55	14	-
31	Kim et al. (2012) [42]	87	-	-	-	-	69	18	-
32	Manabe et al. (2011) [43]	37	11	15	11	0	26	11	26
33	Sommer et al. (2011) [44]	40	17	18	-	-	35	5	23
34	Reimann et al. (2010) [45]	23	-	-	-	-	11	12	
35	Schmidt et al. (2009) [46]	23	5	6			11	12	18
36	Ejaz et al. (2006) [47]	-	-	-	-	-	-	-	-
37	Sigurdardóttir et al. (2001) [48]	-	-	-	-	-	-	-	-
38	Plaschke et al. (1998) [49]	-	-	-	-	-	-	-	-
39	Senard et al. (1992) [50]	-	-	-	-	-	-	-	-
40	Micieli et al. (1989) [51]	-	-	-	-	-	-	-	-
	TOTAL	2619	477 / 1179 (40.45%)	310 / 869 (35.67%)	106 / 451 (23.5%)	23 / 451 (5.09%)	1744 / 2309 (75.53%)	565 / 2309 (24.46%)	702 / 1179 (59.54%)

Table S3. Blood preasure measurements.

No.	Study	No. of Patients	Mean 24-hour SBP/DBP (mmHg)	Mean Diurnal SBP/DBP (mmHg)	Mean Nocturnal SBP/DBP (mmHg)
1	Arici and Helvaci (2020) [15]	35	122.6 ± 10.5 / 75.1 ± 6.5	-	-
2	Chen et al. (2020) [16]	101	120.2 ± 12.7 / 72.0 ± 8.6	119.7 ± 12.7 / 72.1 ± 8.5	121.2 ± 17.8 / 71.1 ± 11.0

3	Di Stefano et al. (2020) [17]	52	122.1 / 72.8	120.9 / 73.3	123.9 / 71.3
4	Oka et al. (2020) [18]	75	-	-	-
5	Yoo et al. (2020) [19]	97	114.3 ± 11.9 / 71.3 ± 9.4	115.1 ± 12.4 / 71.8 ± 9.6	111.6 ± 12.9 / 68.5 ± 11.6
6	Kotagal et al. (2019) [12]	35	129.8 / 73.1	-	123.7 / 73
7	Li et al. (2019) [20]	150	115.9 / 73.3	-	-
8	Vallelonga et al. (2019) [21]	113	-	121 / 73.5	115.6 / 66.3
9	Vallelonga et al. (2019) [22]	72	-	120 ± 13 / 72 ± 8	131 ± 18 / 75 ± 12
10	Milazzo et al. (2018) [23]	114	121 ± 10 / 72 ± 7	122 ± 10 / 74 ± 8	117 ± 15 / 68 ± 10
11	Tanaka et al. (2018) [24]	137	115.1 ± 12 / 75.8 ± 8.4	116.8 ± 11.9 / 78.2 ± 8.9	113 ± 15.1 / 72.2 ± 10
12	Yamashiro et al. (2018) [25]	128	115.3 / 75.7	116.9 / 78	113.4 / 72.2
13	Franzen et al. (2017) [26]	27	-	125.6 ± 13.6 / 74.8 ± 8.2	123.1 ± 14.8 / 73.1 ± 9.9
14	Kanegusuku et al. (2017) [27]	21	120 ± 2 / 73 ± 2	121 ± 2 / 75 ± 2	117 ± 3 / 69 ± 2
15	Kim et al. (2017) [28]	125	-	-	113 ± 15.4 / -
16	Kim et al. (2017) [29]	99	-	-	114.1 ± 14.7 / -
17	Vetrano et al. (2017) [30]	167	-	-	-
18	Vichayanrat et al. (2017) [31]	51	124.2 / 74.5	125.2 / 75.5	119.2 / 68.7
19	Kang et al. (2016) [32]	46	126.1 ± 14 / 77.5 ± 8.7	127.3 ± 12.9 / 78.2 ± 7.6	122.7 ± 19.7 / 74.2 ± 12.3
20	Kim et al. (2016) [33]	188	-	-	112.4 / -
21	Park et al. (2016) [34]	129	-	-	-
22	Stuebner et al. (2015) [35]	21	-	125 ± 11 / 86 ± 12	117 ± 14 / 79 ± 14
23	Vetrano et al. (2015) [36]	125	120.4 / 72.6	120.5 / 73.1	120 / 70.2
24	Fanciulli et al. (2014) [14]	16	-	126 / 71	112 / 65
25	Kim et al. (2014) [37]	65	-	-	110.2 / -
26	Oh et al. (2014) [38]	225	-	-	-
27	Pilleri et al. (2014) [13]	61	123.9 ± 13.3 / 78.8 ± 8.3	122.9 ± 13.6 / 78.9 ± 8.8	128.4 ± 20.4 / 78.5 ± 11.3
28	Berganzo et al. (2013) [39]	111	122.9 ± 16.4 / 73.8 ± 9.8	124.1 ± 19.8 / 75.6 ± 12.1	118.8 ± 17.7 / 69.3 ± 10.4
29	Oh et al. (2013) [40]	129	-	116.3 ± 11.9 / 71.7 ± 10.2	110.5 ± 13.6 / 67.7 ± 11.3
30	Oh et al. (2013) [41]	69	-	-	-
31	Kim et al. (2012) [42]	87	-	-	-
32	Manabe et al. (2011) [43]	37	130 ± 14.8 / 74.9 ± 7.8	130.8 ± 14.1 / 75.2 ± 7.7	125.5 ± 22.2 / 72.7 ± 12.7
33	Sommer et al. (2011) [44]	40	130.3 / 77.6	131.4 / 78.7	129.6 / 75.4
34	Reimann et al. (2010) [45]	26	-	-	-
35	Schmidt et al. (2009) [46]	23	138.5 ± 17.2 / 81.9 ± 10.2	140.6 ± 19.3 / 84 ± 11	131 ± 21.3 / 75.6 ± 12.3
36	Ejaz et al. (2006) [47]	13	-	132.1 ± 11 / 77 ± 7.4	151.5 ± 17.7 / 82.5 ± 12.8
37	Sigurdardóttir et al. (2001) [48]	10	121 / 67	122 / 66	122 / 67
38	Plaschke et al. (1998) [49]	24	-	119.8 / -	-
39	Senard et al. (1992) [50]	38	125.5 / 76.5	123.5 / 74.5	127 / 75.5
40	Micieli et al. (1989) [51]	5	157 ± 68.9 / 75.6 ± 9.4	-	-

SBP = systolic blood pressure, DBP = diastolic blood pressure.
